# Preliminary Report Concerning the Passage of Bacteria through the Tonsillar Tissue as Determined by Experimental Research

**Published:** 1913-10

**Authors:** George B. Wood

**Affiliations:** Philadelphia


### Preliminary Report Concerning the Passage of Bacteria Through the Tonsillar Tissue as Determined by Experimental Research.

Dr. George B. Wood, Philadelphia: From a study of our experiments it seems that the following conclusions may be drawn: 1. The tonsils in the hog are more readily infected by the anthrax bacillus than any other portion of the buccal or pharyngeal mucosa. The clinical history of this disease in the hog shows that in the great majority of idiopathic cases the pharynx has been the site of invasion, and in all of these cases of pharyngeal diseases the tonsils are the port of entry. In none of my experiments was there any involvement of the pharyngeal or buccal mucosa other than the tonsils. While the culture of anthrax was generally brought into more intimate contact with one of the tonsils, it was impossible to limit the bacilli to the tonsillar surface and they came into contact with a large part of the pharynx. In the infection an effort was made to rub the emulsion into one tonsil only and in one case the lesions were limited to one tonsil only, but this was not the tonsil on which the culture had been rubbed. 2. Anthrax bacilli penetrate through the cryptical and not the surface of the epithelium. 3. The anthrax bacillus probably gains access to the parenchyma of the tonsils by passing through the living unaltered epthelium, and having gained access through the superficial layers of the epithelium, they tended to multiply in the deeper layers and then pass into the interfollicular tissue. 4. The anthrax bacilli penetrating through the living normal epithelium cause a devitalization of the tissue, which paves the way for secondary infection from the staphylococci or other pathogenic organisms. 5. The rapidity of the invasion is influenced both by virulence of the organism and the susceptibility of the individual animal. Following the invasion the subsequent course of the disease is similar to that found in other tissues. The toxin elaborated by the bacilli causes at first an accumulation of polymorphonuclear cells, later necrosis of the tissue cells with disintegration of the nuclei. The germinating follicles show more resistance to the disease than the interfollicular tissue. Associated with the necrotic process is an increase in the number and engorgement of the capillaries, and sometimes there is marked extravasation of the red blood cells The anthrax bacilli accumulate in the lymph spaces and also around the blood vessel walls. In some of the sections examined the bacilli were found penetrating the blood vessel walls, and a few were found actually in the blood current.

